# Multifunctional Tyrosinase Inhibitor Peptides with Copper Chelating, UV-Absorption and Antioxidant Activities: Kinetic and Docking Studies

**DOI:** 10.3390/foods10030675

**Published:** 2021-03-22

**Authors:** Pei-Gee Yap, Chee-Yuen Gan

**Affiliations:** Analytical Biochemistry Research Centre, University Innovation Incubator Building, Universiti Sains Malaysia, Lebuh Bukit Jambul, Bayan Lepas 11900, Penang, Malaysia; peggy-yap@hotmail.com

**Keywords:** anti-pigmentation peptide, anti-aging, structure-activity relationship analysis, metal chelation, sun protection factor

## Abstract

Nature-derived tyrosinase inhibitors are of great industrial interest. Three monophenolase inhibitor peptides (MIPs) and three diphenolase inhibitor peptides (DIPs) from a previous study were investigated for their in vitro tyrosinase inhibitory effects, mode of inhibition, copper-chelating activity, sun protection factor (SPF) and antioxidant activities. DIP1 was found to be the most potent tyrosinase inhibitor (IC_50_ = 3.04 ± 0.39 mM), which could be due to the binding interactions between its aromatic amino acid residues (Y2 and D7) with tyrosinase hotspots (H85, V248, H258, H263, F264, R268, V283 and E322) and its ability to chelate copper ion within the substrate-binding pocket. The conjugated planar rings of tyrosine and tryptophan may interact with histidine within the active site to provide stability upon enzyme-peptide binding. This postulation was later confirmed as the Lineweaver–Burk analysis had identified DIP1 as a competitive inhibitor and DIP1 also showed 36.27 ± 1.17% of copper chelating activity. In addition, DIP1 provided the highest SPF value (11.9 ± 0.04) as well as ferric reducing antioxidant power (FRAP) (5.09 ± 0.13 mM FeSO_4_), 2,2′–azinobis(3-ethylbenzothiazoline-6-sulphonic acid) diammonium salt (ABTS) (11.34 ± 0.90%) and 2,2-diphenyl-1-picrylhydrazyl (DPPH) (29.14 ± 1.36%) free radical scavenging activities compared to other peptides. These results demonstrated that DIP1 could be a multifunctional anti-tyrosinase agent with pharmaceutical and cosmeceutical applications.

## 1. Introduction

Tyrosinase belongs to the type 3 copper protein family which catalyses the *ortho*-hydroxylation of phenolic substrates (monophenolase reaction) and the two-electron oxidation of catecholic substrates (diphenolase reaction) to quinones. The subsequent oxidation and polymerization of quinones leads to the formation of melanin, which is responsible for hair, skin and eye pigmentation in human and animals. This ubiquitous pigment plays a crucial role in photoprotection by forming supranuclear melanin caps in keratinocytes to protect the cells against ultraviolet (UV)-induced DNA damages and mutations [1]. This is because melanin absorbs light across a wide spectrum and then effectively dissipates the absorbed energy into heat endowed by its highly conjugated molecular structure [2]. Hence, melanin acts as a natural sunscreen to hinder the penetration of UV through the skin epidermis to protect against DNA damages. However, the overproduction of melanin i.e., hyperpigmentation is associated with skin pigment disorders including café au lait macules, melasma, freckles and age spots [3,4,5].

Effective tyrosinase inhibitors such as kojic acid and hydroquinone have been long discovered, yet each suffered from different side effects which hindered their applications as depigmentation agents. For instance, kojic acid is associated with erythema, stinging sensations, mild exfoliation and contact eczema [6]. Hydroquinone received a controversial reputation for health and safety reasons since it is a metabolite of benzene, a known carcinogen. The long-term accumulation of hydroquinone is also associated with ochronosis, nephrotoxicity and melanocyte toxicity [7]. Thus, natural-sourced tyrosinase inhibitors such as peptides are of great potential due to their relatively low toxicity and specific in action.

In our previous research, egg white protein hydrolysate was found to inhibit tyrosinase activity and the subsequent bioinformatics-aided screening had identified a number of monophenolase and diphenolase inhibitory peptides [8]. In particular, three monophenolase inhibitor peptides (MIPs) and three diphenolase inhibitor peptides (DIPs) were found to have potential in inhibiting tyrosinase. Therefore, this study focused on the identification of the mode of inhibition of these six selected peptides. Further delineation of tyrosinase-peptide binding interactions through structure–activity relationship (SAR) analysis can nevertheless provide a more comprehensive understanding of the peptide mechanism of action. The copper-chelating activity of the peptides was also of interest as reports had related tyrosinase inhibitory activity with the ability to chelate copper ions [9]. Other than that, UV protection by melanin would be reduced if a peptide inhibits tyrosinase because tyrosinase is the rate-limiting enzyme for melanin production. Hence, it would be beneficial for the peptide inhibitor to provide a sunscreen effect in order to complement for the loss of melanin protection. The antioxidant effect could also be a bonus property of the peptide inhibitor to provide anti-ageing effects to consumers. Therefore, the objectives of this study were (i) to determine the mode of inhibition, (ii) to elucidate the peptide-binding interactions with tyrosinase, and to determine their (iii) copper-chelating activity, (iv) sun protection factor (SPF) and (v) antioxidant activities.

## 2. Materials and Methods

### 2.1. Peptide Synthesis

Three monophenolase inhibitor peptides (MIPs) and three diphenolase inhibitor peptides (DIPs) were chemically synthesized by Medigene, Malaysia. The MIPs ADHPF (MW = 585.6 Da), ILELPFASGDLLML (MW = 1531.87 Da) and FDKLPGFGD (MW = 995.08 Da) were denoted as MIP1, MIP2 and MIP3 whereas the DIPs GYSLGNWVCAAK (MW = 1268.44 Da), HIATNAVLFFGR (MW = 1345.56 Da) and FMMFESQNKDLLFK (MW = 1778.11 Da) were denoted as DIP1, DIP2 and DIP3 respectively. The molecular weights of the synthesized peptides were determined using mass spectrometry analysis and their purities were higher than 95% according to the high-performance liquid chromatography (HPLC) result from Medigene, Malaysia.

### 2.2. Chemicals

Mushroom tyrosinase (7164 U/mg), ʟ-tyrosine, ʟ-DOPA, pyrocatechol violet and 2,2′–azinobis(3-ethylbenzothiazoline-6-sulphonic acid) diammonium salt (ABTS) were purchased from Sigma-Aldrich whereas 2,4,6-tris(2-pyridyl)-s-triazine (TPTZ) and 2,2-diphenyl-1-picrylhydrazyl (DPPH) were purchased from Fluka. Copper (II) sulphate-5-hydrate was purchased from Bendosen. Other chemicals used in this study were purchased from Sigma-Aldrich and were of analytical grade.

### 2.3. Determination of Tyrosinase Inhibitory Activities

The monophenolase and diphenolase inhibitory activities of tyrosinase were determined by using ʟ-tyrosine and ʟ-DOPA as substrates, respectively, as previously described [8]. Various concentrations of peptides were tested to determine the IC_50_ values, i.e., the concentration of peptides required to inhibit half of the tyrosinase activities.

### 2.4. Determination of Mode of Inhibition

The activities of monophenolase and diphenolase reactions were determined through the measurement of the rate of formation of dopachrome in the presence of substrate concentrations, [S] ranging from 0.125 to 0.5 mM. The initial velocity, V of the reaction was determined using the Beer–Lambert law:

(1) ΔA=ε l Δc

(2) $$V=\frac{\Delta C}{t}=\frac{\Delta A}{\varepsilon \;l\;t}$$

where $\frac{\Delta C}{t}$ denotes the rate of change of the solution concentration; $\frac{\Delta A}{t}$ denotes the rate of change of absorbance i.e., slope of the reaction; ε denotes the molar extinction coefficient of dopachrome (3700 M^−1^ cm^−1^) and l denotes the pathlength.

The mode of inhibition of peptides was subsequently determined using the Lineweaver–Burk plot of $\frac{1}{V}$ against $\frac{1}{\left[S\right]}$ according to the point of intersection of the curves in the presence of different peptide concentrations.

### 2.5. Determination of Copper Chelating Activity

The copper chelating activity was determined according to Kubglomsong et al. [9]. Briefly, 4 mM of chromogenic reagent, i.e., pyrocatechol violet was prepared in 50 mM sodium acetate buffer (pH 6.0). Then, 10 µL of peptide sample (10 mg/mL), 280 µL sodium acetate buffer, 10 µL CuSO_4_∙5H_2_O and lastly 6 µL pyrocatechol violet were added to a 96-well plate. The sample solution was mixed well and the absorbance was measured at 632 nm using a spectrophotometer (Spectramax M5, Molecular Devices, San Jose, CA, USA). Ethylenediaminetetraacetic acid (EDTA) was used as the positive control. The copper chelating activity is calculated as follows:

(3) $$Copper\;chelating\;activity\;\left(\%\right)=\frac{A_{control}-A_{sample}}{A_{control}}\times 100$$

where *A_control_* denotes the absorbance of the system containing sodium acetate buffer, pyrocatechol violet and CuSO_4_∙5H_2_O whereas *A_sample_* denotes the absorbance of the system containing peptide, sodium acetate buffer, pyrocatechol violet and CuSO_4_∙5H_2_O.

### 2.6. Structure–Activity Relationship (SAR) Analysis

The PepFold3 web server [10] was used to generate the three-dimensional structures of the selected peptides [11]. One hundred independent predictions of peptide structure were executed using the default settings according to the peptide amino acid sequence provided. The generated peptide structure with the lowest coarse-grained optimized potential for efficient structure prediction (sOPEP) energy was selected for molecular docking.

The X-ray crystal structure of mushroom tyrosinase (PDB ID: 2Y9X) was obtained from the Protein Data Bank (PDB) [12]. Addition of polar hydrogens was performed using the AutoDock 4.2 software and a +2 charge was assigned to the copper ions [13]. The HADDOCK 2.2 webserver [14] was employed to predict the protein–peptide binding conformations [15]. In the HADDOCK submission form, the tyrosinase and peptide structure files were uploaded as Molecule 1 and 2, respectively. The identity of tyrosinase active residues predicted to make contact with peptide were provided according to the preliminary screening results [8]. Rigid molecular docking with flexibility on both active protein side chains and peptide structures were executed using the default settings. The protein–peptide docking solutions were clustered and ranked by HADDOCK score, i.e., cluster with the lowest HADDOCK score is the most likely conformation. Subsequently, the PRODIGY web server [16] was used to predict the binding affinity of the protein–peptide complex [17]. The visualization of the protein–peptide binding interactions was conducted using the LigPlot+ v.2.2. software.

### 2.7. Determination of Sun Protection Factor (SPF)

The ability of peptides to absorb light in the UVB region (290–320 nm) was measured according to the methods described by Dutra, Kedor-Hackmann and Santoro [18]. The UV–Vis spectrophotometer was set to scan the peptide samples from 220 to 400 nm with a 5 nm increment. The SPF values of the peptide samples (10 mg/mL) were calculated using the Mansur equation:

(4) $$SPF=CF\times \overset{320}{\underset{290}{\sum }}EE\left(\lambda \right)\times I\left(\lambda \right)\times Abs\left(\lambda \right)$$

where *CF* denotes correction factor of 10 (for the short pathlength used); *EE* denotes erythemal effect spectrum; *I* denotes solar intensity spectrum and *Abs* denotes the absorbance of sample.

The values of EE×I were normalized to 1 by Sayre, Agin, LeVee and Marlowe [19] i.e. the EE×I values for wavelengths 290, 295, 300, 305, 310, 315 and 320 were 0.0150, 0.0817, 0.2874, 0.3278, 0.1864, 0.0839 and 0.0180, respectively.

### 2.8. Determination of Antioxidant Activities

#### 2.8.1. Ferric Reducing Antioxidant Power (FRAP) Assay

The reducing potential of peptides was measured according to Benzie and Strain [20]. ferric reducing antioxidant power (FRAP) reagent containing 300 mM acetate buffer (pH 3.6), 10 mM TPTZ prepared in 40 mM HCl and 20 mM FeCl_3_⸱6H_2_O in the ratio of 10:1:1 was incubated at 37 °C for 30 min. Then, 200 µL of FRAP reagent was added to 2.7 µL peptide sample (10 mg/mL) in a 96-well plate and mixed well before incubating at 37 °C for 1 h. The absorbance of the sample was then measured spectrophotometrically at 593 nm. Freshly prepared FRAP reagent was used for each run. A standard curve was prepared using FeSO_4_⸱7H_2_O at 0–3 mM. The peptide FRAP was expressed in mM FeSO_4_. Gallic acid was used as the positive control.

#### 2.8.2. 2,2′-Azinobis(3-ethylbenzothiazoline-6-sulphonic acid) Diammonium Salt (ABTS) Free Radical Scavenging Assay

The free radical scavenging potential of peptides was measured according to Cai, Luo, Sun and Corke [21]. Briefly, ABTS reagent containing 7 mM ABTS and 2.45 mM ammonium persulfate were incubated in the dark at room temperature for 12–16 h. The reagent was diluted using 80% ethanol to an absorbance of 0.700 ± 0.005 at 734 nm. Then, 195 µL of the diluted ABTS reagent was added to 5 µL of peptide sample (10 mg/mL) and incubated in the dark at room temperature for 6 min. The absorbance of the sample at 734 nm was monitored immediately using a spectrophotometer. Gallic acid was used as the positive control. The ABTS free radical scavenging activity is calculated as follows:

(5) $$ABTS\;free\;radical\;scavenging\;activity\;\left(\%\right)=\frac{A_{control}-A_{sample}}{A_{control}}\times 100$$

where *A_control_* denotes the absorbance of the system containing *ABTS* and water whereas *A_sample_* denotes the absorbance of the system containing *ABTS* and peptide sample.

#### 2.8.3. 2,2-Diphenyl-1-picrylhydrazyl (DPPH) Free Radical Scavenging Assay

The free radical scavenging potential of peptides was measured according to Blois [22]. Briefly, 0.1 mM DPPH stock solution was prepared in ethanol. In a dark setting, 500 µL of DPPH was added to 16.65 µL peptide sample (10 mg/mL) and incubated at 30 °C for 30 min. The absorbance of the mixture was then monitored at 517 nm using a spectrophotometer. Gallic acid was used as the positive control. The DPPH free radical scavenging activity is calculated as follows:

(6) $$DPPH\;free\;radical\;scavenging\;activity\;\left(\%\right)=\frac{A_{control}-A_{sample}}{A_{control}}\times 100$$

where *A_control_* denotes the absorbance of the system containing *DPPH* and ethanol whereas *A_sample_* denotes the absorbance of the system containing *DPPH* and peptide sample.

### 2.9. Statistical Analysis

Statistical analysis was conducted using SPSS version 20.0 (SPSS Institute, Chicago, IL, USA). The results were analysed using one-way ANOVA followed by Duncan’s test. *p*-value < 0.05 implies a significant difference between the sample means. Three independent experiments were performed in triplicates each and the results were reported as mean ± standard deviation.

## 3. Results and Discussion

### 3.1. Tyrosinase Inhibitory Activities

**Figure 1:**
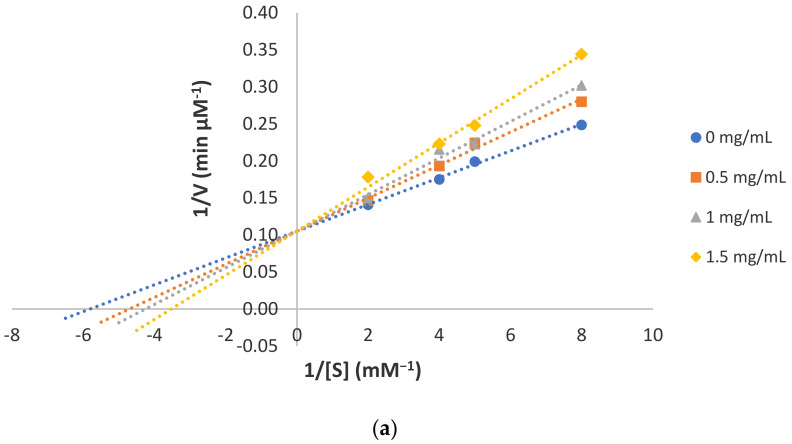

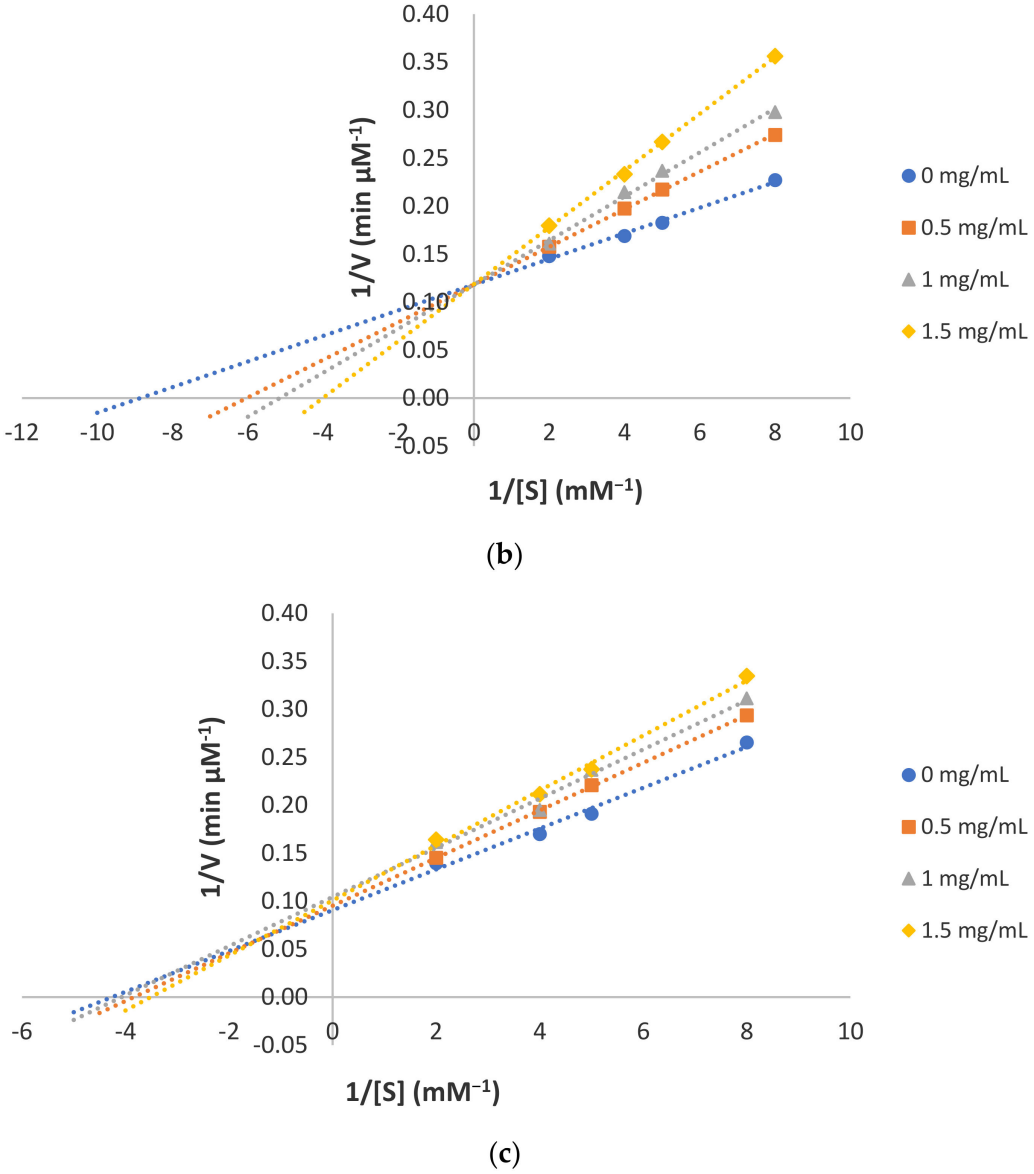
Lineweaver–Burk plot for the inhibition of monophenolase activity by (**a**) MIP1; (**b**) MIP2; and (**c**) MIP3.

The effects of MIPs and DIPs on the monophenolase and diphenolase activities of tyrosinase were shown in Table 1. For monophenolase reaction (Table 1a), the highest inhibition was achieved by MIP1 (35.86 ± 2.26%). The Lineweaver–Burk plot showed straight lines intersecting at the same y-intercept when the peptide concentration increased from 0 to 1.5 mg/mL (Figure 1), suggesting MIP1 as a competitive inhibitor of monophenolase reaction. Then, MIP2 was a weaker competitive inhibitor compared to MIP1 as it only inhibited 17.26 ± 2.10% of monophenolase reaction (Table 1a). Notably, most monophenolase inhibitory peptides with IC_50_ < 10 µM were found to comprise of only 2–3 residues [23,24]. Thus, long-chained peptide may not be a favourable characteristic for monophenolase inhibition. In contrast, MIP3 showed 22.78 ± 1.12% inhibition of monophenolase activity (Table 1a). The Lineweaver–Burk plot showed the intersection of straight lines with different slopes at the second quadrant (Figure 1), suggesting MIP3 as a mixed-type inhibitor. MIP3 may bind to the tyrosinase allosteric site to induce irreversible modification of catalytic site conformation and subsequently reduce substrate binding. Examples of tyrosinase mixed-type inhibitor include p-coumaric acid and 3-amino-ʟ-tyrosine with IC_50_ values < 0.36 mM and 14 µM, respectively [25,26].

**Figure 2:**
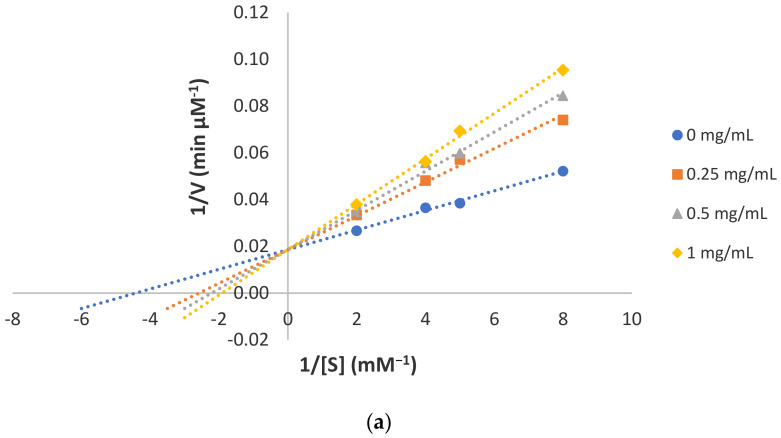

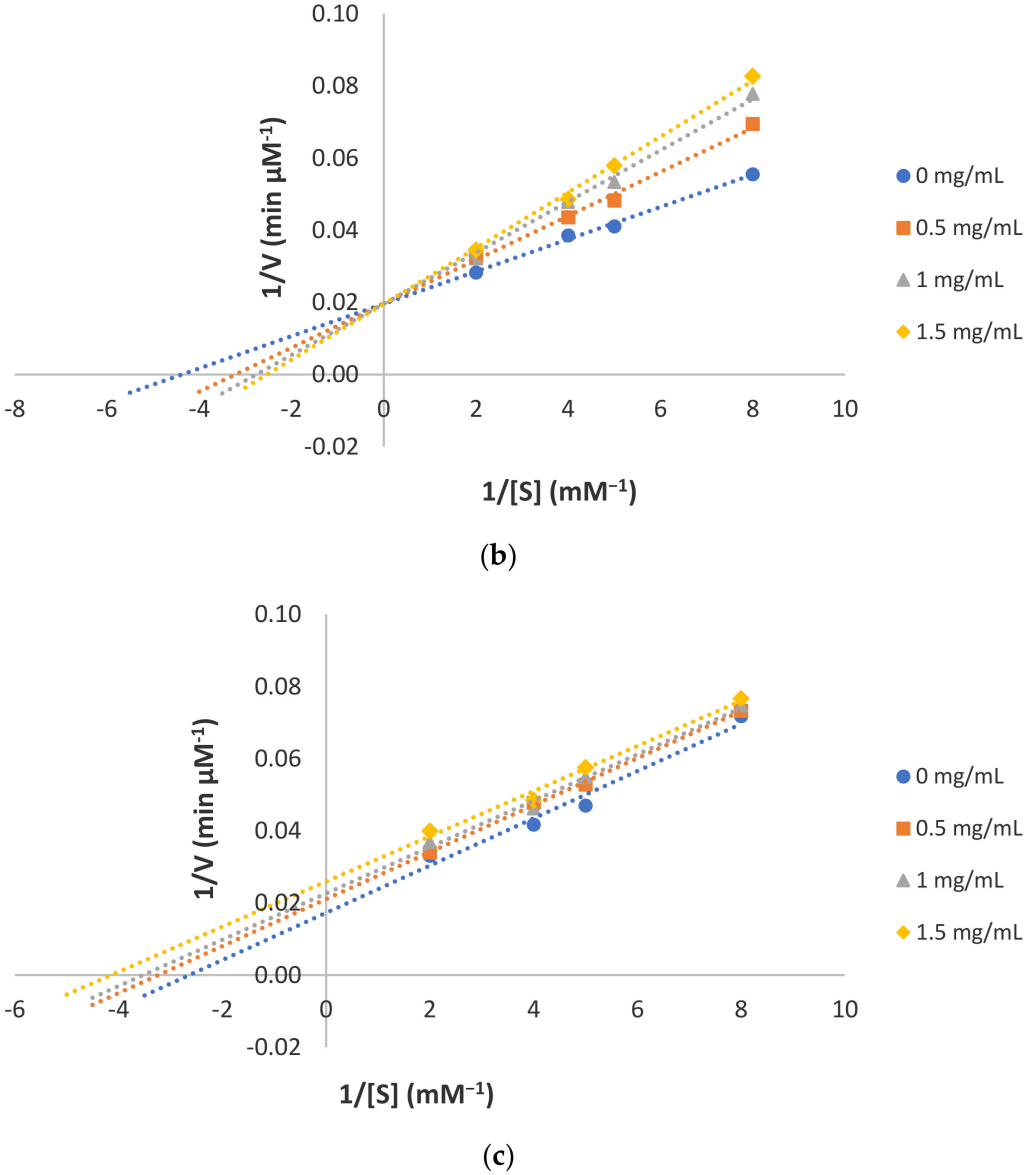
Lineweaver–Burk plot for the inhibition of diphenolase activity by (**a**) DIP1; (**b**) DIP2; and (**c**) DIP3.

The highest diphenolase activity inhibition was recorded by DIP1 (80.04 ± 2.79%) (Table 1b) and the IC_50_ determined was 3.04 ± 0.39 mM. Results from kinetic studies suggested this peptide as a competitive inhibitor (Figure 2). However, DIP2 appeared to be a weaker competitive inhibitor since it only inhibited 17.70 ± 2.66% of diphenolase activity (Table 1b). In contrast to MIP1 and MIP2, the length and molecular weight of DIP1 and DIP2 were similar hence the peptides’ mechanism of inhibition was further investigated using molecular docking (Section 3.3). DIP3, on the other hand, inhibited 28.95 ± 1.47% of diphenolase reaction (Table 1b). The parallel straight lines on the double reciprocal plot suggested DIP3 as an uncompetitive inhibitor of diphenolase reaction (Figure 2). A similar mode of inhibition was reported for the Cl^-^ ion and the bipyridine compounds where the authors hypothesized that the inhibitor binding sites were induced due to conformational changes upon a substrate binding to the enzyme [27,28].

### 3.2. Copper Chelating Activity

The binuclear copper atoms are essential for the catalytic activities of tyrosinase, hence copper chelation may suppress enzyme activity. Based on Table 1, the highest copper chelating activity was recorded by MIP1 (94.33 ± 0.04%) and MIP3 (94.43 ± 0.46%) followed by DIP1 (36.27 ± 1.66%). According to Torres-Fuentes, Alaiz and Vioque [29], low molecular weight peptides (0.1–1.2 kDa) were stronger copper chelators. Our result coincided with this finding as the molecular weights of the three peptides ranged between 0.6–1.3 kDa and the weights of MIP1 < MIP3 < DIP1. Carrasco-Castilla et al. [30] suggested that the peptide charge and acidity may be crucial for copper chelation. Thus, for MIPs, the high activity of MIP1 and MIP3 may be contributed by the relative higher composition of aspartic acid residue in the peptide sequence which is capable of interacting with copper via its carbonyl group. However, MIP1 and MIP3 showed modest inhibition against the monophenolase reaction of tyrosinase, suggesting an alternative inhibitory mechanism independent of copper chelation. Both peptides may act as reducing agents for tyrosinase inhibition [31]. This is because MIP1 contains an aspartic acid residue whereas MIP3 contains aspartic acid and lysine residues in their sequences. The charged amino acids possess hydrogen donor atoms in their side chains which can donate hydrogen to the carbonyl oxygen in *o*-dopaquinone functional group. The acceptance of hydrogen reduces *o*-dopaquinone back to ʟ-DOPA hence hindering the subsequent melanogenesis steps. On the other hand, the high copper chelating activity of DIP1 was accompanied by high diphenolase inhibition (Table 1b), which could be due to the presence of aromatic amino acids such as tyrosine and tryptophan within the peptide sequence. The phenolic and indolic structures of tyrosine and tryptophan side chains were hypothesized to form a coordination complex with copper ion in the catalytic site through π-cation interaction [32]. The positively charged imidazole ring of histidine surrounding the copper ions may further participate in proton transfer with the indole group of tryptophan to stabilize the enzyme–peptide structure [33]. Therefore, structural and electronic properties of peptide side chains may play important roles in the resultant copper chelating and tyrosinase inhibitory effects.

### 3.3. Structure–Activity Relationship (SAR) Analysis

Mushroom tyrosinase is a tetrameric protein made up of two light chains and two heavy chains. The heavy chains form the enzyme active site whereas the light chains are not catalytically important [34]. The enzyme active site resembles a tunnel which is comprised of three regions i.e., a solvent-exposed opening, a hydrophobic tunnel and an inner substrate-binding pocket [13]. The hotspots of the solvent-exposed region include the hydrophilic residues E189 and R268, whereas the hotspots of the hydrophobic tunnel include V248, F264, V283 and P284. The innermost flexible substrate-binding pocket houses two catalytically crucial copper atoms denoted as CuA and CuB and six highly conserved histidine residues surrounded by the charged residues E256 and E322 [34]. Phenolic substrates (e.g., tyrosine) bind to CuA coordinated by H61, H85, H94 during monophenolase reaction whereas catecholic substrates (e.g., ʟ-DOPA) bind to CuB coordinated by H259, H263 and H296 during diphenolase reaction [35].

**Figure 3:**
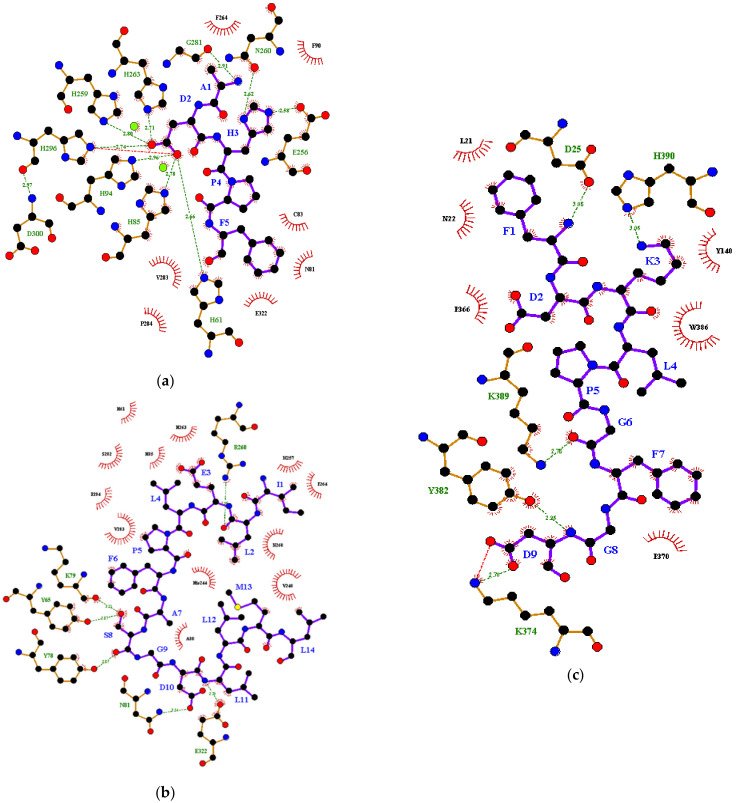
Binding interactions between (**a**) MIP1, (**b**) MIP2, (**c**) MIP3 and tyrosinase during the monophenolase reaction. Red ball, oxygen atom; green ball, copper atom; blue ball, nitrogen atom; black ball, carbon atom; yellow ball, cysteine atom; purple line, peptide; brown line, tyrosinase; cyan line, covalent bond; red dotted line, salt bridge; green dotted line with number, hydrogen bonding and the distance (in Armstrong, Å) between the proton donor and acceptor; Brick red eyelashes, hydrophobic interaction.

HADDOCK molecular docking analysis was performed to predict the potential protein–peptide binding interactions followed by the prediction of protein–peptide binding affinity using the PRODIGY server. The protein–peptide binding interactions were summarized in Table 2 and illustrated in Figure 2. For monophenolase reaction (Table 2a), MIP1 was found to occupy a tyrosinase active site with the free energy of binding -9.0 kcal/mol. It formed hydrogen bonds with the substrate-binding pocket hotspots H61, H85, H94, H259, H263, H296 as well as hydrophobic interactions with the two copper ions via its D2 residue (Figure 3), which may disturb the homolytic dissociation of the product molecule from tyrosinase during the final step of monophenolase reaction [36]. Although MIP2 was found to interact with tyrosinase hotspots in a similar way to MIP1, no binding interaction with copper ions was present (Figure 3). This is in accordance to the experimental results where MIP2 showed significantly (*p* < 0.05) lower monophenolase inhibition and copper chelating activities than MIP1. The large molecular weight and bulky side chains of MIP2 may reduce its diffusion towards the active site due to large steric hindrance. On the other hand, MIP3 did not interact with any of the tyrosinase hotspots and was bound to a groove at the back of the enzyme active site with a binding energy of −7.0 kcal/mol (Table 2a). This binding site was adjacent to the binding sites occupied by the mixed-type inhibitors phthalic acid and cinnamic acid, as reported by Hassani, Haghbeen and Fazli [37]. Notably, a salt bridge interaction was formed between the anionic carboxylate of MIP3 D9 residue and the cationic ammonium of tyrosinase K374 residue (Figure 3). The non-covalent salt bridge interaction may influence enzyme conformation, stability or substrate recognition at the catalytic site.

**Figure 4:**
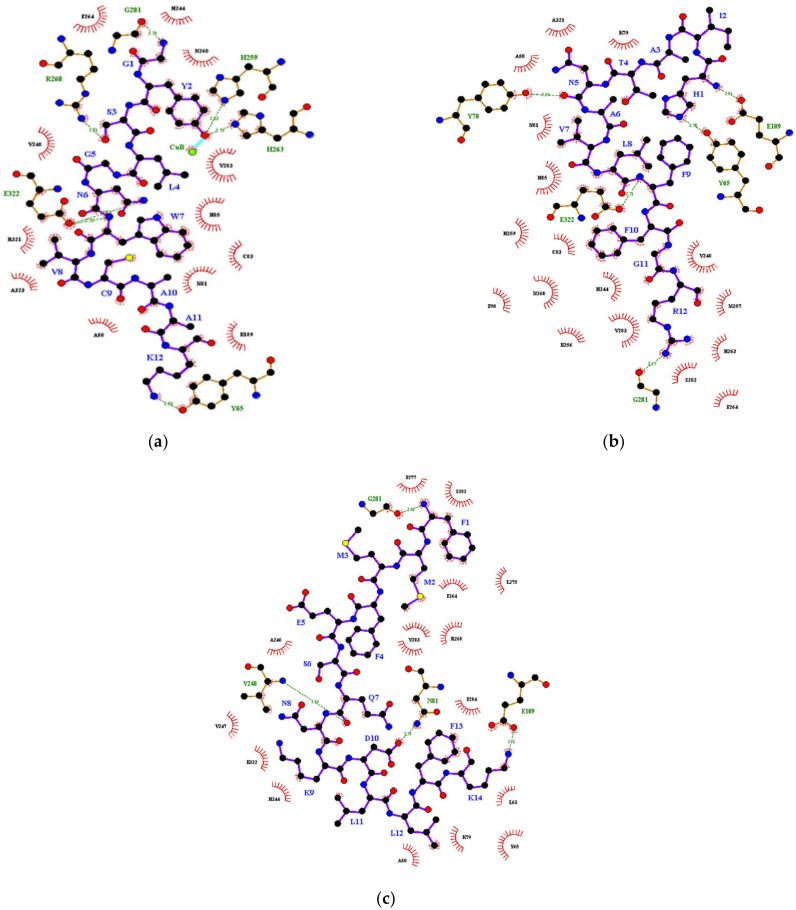
Binding interactions between (**a**) DIP1, (**b**) DIP2, (**c**) DIP3 and tyrosinase during the diphenolase reaction. Red ball, oxygen atom; green ball, copper atom; blue ball, nitrogen atom; black ball, carbon atom; yellow ball, cysteine atom; purple line, peptide; brown line, tyrosinase; cyan line, covalent bond; red dotted line, salt bridge; green dotted line with number, hydrogen bonding and the distance (in Armstrong, Å) between proton donor and acceptor; brick red eyelashes, hydrophobic interaction.

For diphenolase reaction (Table 2b), the lowest free energy of binding −9.4 kcal/mol was achieved by DIP1. The N-terminal penultimate tyrosine residue of DIP1 formed a covalent bond with CuB and two hydrogen bonds with H259 and H263. The W7 residue of DIP1 also hydrophobically interacted with the C83 and H85 of tyrosinase (Figure 4). Aromatic residues in DIP1 seemed crucial to interact with tyrosinase hotspots. In fact, the presence of tyrosine, tryptophan and phenylalanine is a common feature of strong tyrosinase inhibitor peptides, as the aromatic ring may serve as a pseudo substrate or a copper chelator to abolish tyrosinase activity [38,39]. The prediction result supported with the experimental outcome in which DIP1 showed the highest diphenolase inhibitory and copper chelating activities. In contrast, no interaction was predicted between DIP2 and copper ions (Table 2b; Figure 4), suggesting the ability of peptide to chelate copper may be crucial in diphenolase inhibition. Then, DIP3 did not interact with any of the histidine hotspots and was found partially blocking the hydrophobic tunnel leading to the enzyme active site (Figure 4). Hence, the uncompetitive inhibitor DIP3 may bind to the enzyme right after the binding of ʟ-DOPA to the active site. The formation of the enzyme–substrate–inhibitor complex inhibits the catechol cycle and the subsequent catalysis steps. Overall, it was observed that the long-chained peptides DIP1 and DIP2 interacted more frequently with the hotspots H85, H259, H263 instead of H61, H94 and H296. The 5-mer peptide MIP1 was the only peptide capable of interacting with the six histidine residues within the substrate-binding pocket. Similar results were also reported by Shen, Wang, Guo, Tan and Zhang [40]. This is because H85, H259, H263 lay nearer to the tunnel entrance to the binuclear copper active site whereas H61, H94 and H296 were located deep within the tunnel [13]. The inability of DIP1 and DIP2 to interact with all six histidine residues, in contrast to MIP1, could be due to their relatively larger size. The bulky side chains of DIP1 and DIP2 may not be favourable for binding interactions with the histidine hotspots found deep within the substrate-binding pocket. Instead, the side chains of DIP1 and DIP2 were found more frequently to interact with the hydrophilic residues E189 and R268 as well as the hydrophobic tunnel residues V248, F264, V283 and P284, which may aid their diffusion into the binuclear copper site. Therefore, the prediction outcome further confirmed the experimental result in which MIP1, MIP2, DIP1 and DIP2 were competitive inhibitors whereas MIP3 was a mixed-type inhibitor and DIP3 was an uncompetitive inhibitor of tyrosinase.

### 3.4. Sun Protection Factor

**Figure 5:**
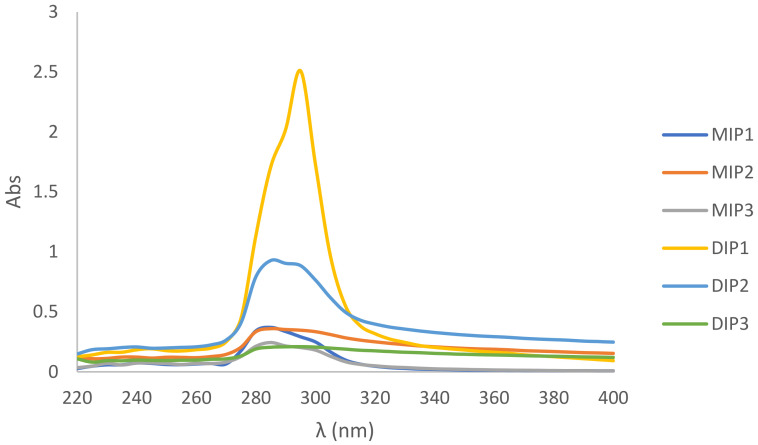
UV–Vis absorption spectra of MIPs and DIPs.

UVB is the major radiation responsible for sunburn and it has a wavelength of 290–320 nm. An effective sunscreen is thus expected to absorb radiation in this region, which is indicated by the SPF value. Based on Figure 5, all MIPs and DIPs showed an absorption peak around 290–300 nm. The highest absorption peak was recorded by DIP1, which had an SPF of 11.9 (Table 1b). The strong UV absorbance may be due to the presence of tryptophan and tyrosine residues within the peptide sequence, which was not found in other peptides. When an electron absorbs a photon, it is excited and tends to move to a higher energy orbital. However, the excited electron is unstable and tends to lose energy in the form of light. The conjugated π-bond system that formed the aromatic ring structure of tryptophan and tyrosine stabilizes the excited electrons by resonance to allow their transition to higher energy states. This confers the strong UV absorbance of tryptophan and tyrosine [41]. In nature, the aromatic ring structure of polyphenols also provides photoprotection to plants in this manner. For instance, stilbenes, flavonoids and hydroxycinnamic acid derivatives from fruits, vegetables and teas were found to possess SPFs 7.3–28.8 [42]. Therefore, the structural property of amino acid residues may be attributed to the degree of photoprotection provided by peptides.

### 3.5. Antioxidant Activities

The antioxidant activities of MIPs and DIPs were shown in Table 1. The slight difference in the antioxidant results could be due to the different mechanisms of the assays. The FRAP assay measures the potential of the sample to reduce Fe^3+^-TPTZ to Fe^2+^-TPTZ. On the other hand, ABTS and DPPH assays measure the oxidizing potential of the sample i.e., the ability to donate hydrogen to ABTS and DPPH free radicals. Based on the results, DIP1 recorded the highest antioxidant activities, i.e., FRAP of 5.09 ± 0.129 mM FeSO_4_, 11.34 ± 0.90% ABTS and 29.14 ± 1.36% DPPH free radical scavenging activities compared to other peptides. The relatively high proportion of alanine, leucine, tryptophan, tyrosine and cysteine residues within the sequence of DIP1 may contribute to its ability to deactivate free radicals either through the hydrogen atom transfer (HAT) or single electron transfer (SET) mechanisms [43,44]. Nonetheless, the antioxidant activities of the peptides were lower than that of positive control. Peptides with molecular weight < 3 kDa were associated with higher antioxidant activities [45]. However, this was not observed in our results, as the molecular weight of MIPs and DIPs ranged between 0.5 and 2.0 kDa. Therefore, other factors such as the amino acid sequence, composition, hydrophobicity, hydrogen bonding and electronic property may account for the low antioxidant activities of MIPs and DIPs [44,46].

## 4. Conclusions

Based on the results, MIPs and DIPs showed various degrees of tyrosinase inhibition using different mechanism of actions. The peptides also showed copper chelating activity, antioxidant activity and provided sunscreen effects. Notably, the strong copper chelating activity of MIP1 and MIP3 resulted in only the modest inhibition of monophenolase activity, suggesting an alternative mechanism of inhibition. The large molecular weight peptide (such as MIP2) may not be a favourable characteristic for the inhibition of monophenolase reaction and copper chelation. Similar results were observed for diphenolase peptide inhibitors where DIP2 and DIP3 showed lower inhibitory effects on tyrosinase. DIP1 was found to be the most potent diphenolase reaction peptide inhibitor in which it showed a competitive mode of inhibition towards tyrosinase. The strong inhibitory effect of DIP1 may be attributed to its ability to chelate copper ion within the enzyme catalytic site. In addition, DIP1 also showed the highest UV absorption and antioxidant activities compared to other peptides. The strong activities of DIP1 could be due to the presence of aromatic amino acid residues such as tyrosine and tryptophan within the peptide sequence. The planar ring structures of the peptide side chains may effectively absorb UV or even interact with those from the histidine residue in the tyrosinase catalytic site to confer stability upon the enzyme-peptide binding. Therefore, the enrichment of peptides with aromatic residues such as Tyr, Phe, Pro, His and Trp may provide positive effects to their tyrosinase inhibitory and antioxidant activities [47,48].

The strong tyrosinase inhibitory activity of DIP1 may find potential applications in the cosmeceutical industry as a natural-derived multifunctional anti-tyrosinase agent. However, the transdermal absorption of peptide represents the major challenge for commercialization. The molecular weight of < 500 Da is ideal for skin delivery [49], however, the large molecular weight of DIP1 (MW = 1268.44 kDa) may limit skin penetration. The modification of peptide or the customization of the delivery mechanism is therefore essential to enhance the peptide permeability across the epithelium [50,51]. Moreover, DIP1 could be used in combination with other depigmentation therapies such as chemical peels, microdermabrasion and laser resurfacing as a synergistic treatment. Superficial injuries followed by physical therapies may improve the epithelial absorption of the peptide-based anti-tyrosinase agent. Nonetheless, a more comprehensive cell-based analysis is required before the successful translation of research findings into a clinically proven, commercialized product.

## Figures and Tables

**Table 1:** Summary of the biological activities of (a) monophenolase inhibitory peptides (MIPs) and (b) diphenolase inhibitory peptides (DIPs).

		Peptide Sequence	Tyrosinase Activity Inhibition ^#^ (%)	Mode of Inhibition	Copper Chelating Activity ^#^ (%)	SPF ^#^	Antioxidant Activity ^#^		
							FRAP (mMFeSO4)	ABTS (%)	DPPH (%)
(a)	MIP1	ADHPF	35.86 ± 2.26 ^d^	Competitive	94.33 ± 0.04 ^e^	1.80 ± 0.02 ^b^	0.035 ± 0.004 ^a^	2.90 ± 0.28 ^b^	n.d.
	MIP2	ILELPFASGDLLML	17.26 ± 2.10 ^a^	Competitive	11.41 ± 0.13 ^a^	3.12 ± 0.13 ^c^	n.d.	1.44 ± 0.27 ^ab^	n.d.
	MIP3	FDKLPGFGD	22.78 ± 1.12 ^b^	Mixed	94.43 ± 0.46 ^e^	1.36 ± 0.02 ^a^	0.034 ± 0.002 ^a^	2.11 ± 0.41 ^ab^	n.d.
(b)	DIP1	GYSLGNWVCAAK	80.04 ± 2.79 ^e^	Competitive	36.27 ± 1.17 ^d^	11.9 ± 0.24 ^e^	5.09 ± 0.13 ^b^	11.34 ± 0.90 ^c^	29.14 ± 1.36 ^a^
	DIP2	HIATNAVLFFGR	17.70 ± 2.66 ^a^	Competitive	19.13 ± 2.22 ^b^	6.45 ± 0.40 ^d^	0.018 ± 0.001 ^a^	n.d.	n.d.
	DIP3	FMMFESQNKDLLFK	28.95 ± 1.47 ^c^	Uncompetitive	26.23 ± 1.03 ^c^	1.98 ± 0.05 ^b^	n.d.	0.99 ± 0.56 ^ab^	n.d.
	Gallic acid						16.6 ± 0.36 ^c^	83.14 ± 3.02 ^d^	81.67 ± 0.03 ^b^
	EDTA				96.06 ± 0.03^e^				

^#^ peptide concentration used was 10 mg/mL; n.d., not detected; SPF, sun protection factor; FRAP, ferric reducing antioxidant power; ABTS, 2,2′–azinobis(3-ethylbenzothiazoline-6-sulphonic acid) diammonium salt; DPPH, 2,2-diphenyl-1-picrylhydrazyl; superscript letters in the same column are significantly different (*p* < 0.05) from each other according to Duncan’s test; results were reported as the mean ± standard deviation (n = 3).

**Table 2:** Predicted binding interactions of (a) MIPs and (b) DIPs with tyrosinase.

		Free Binding Energy (kcal/mol)	Hydrophobic Interaction	Salt Bridge	Covalent Bond	Hydrogen Bond
(a)	MIP1	−9.0	CuA	H296	n.d.	H61
			CuB			H85
			N81			H94
			C83			E256
			F90			H259
			F264			N260
			V283			H263
			P284			G281
			E322			H296
	MIP2	−9.5	H61	n.d.	n.d.	Y65
			Y65			Y78
			Y78			K79
			K79			N81
			A80			R268
			N81			E322
			H85			
			V248			
			M257			
			N260			
			H263			
			F264			
			R268			
			S282			
			V283			
			P284			
			E322			
	MIP3	−7.0	L21	K374	n.d.	D25
			N22			K374
			D25			Y382
			Y140			K389
			P366			H390
			P370			
			K374			
			Y382			
			W386			
			K389			
			H390			
(b)	DIP1	−9.4	A80	n.d.	Cu(B)	Y65
			N81			H259
			C83			H263
			H85			R268
			E189			G281
			H244			E322
			V248			
			N260			
			F264			
			V283			
			R321			
			A323			
	DIP2	−8.3	K79	n.d.	n.d.	Y65
			A80			Y78
			N81			E189
			C83			G281
			H85			E322
			F90			
			H244			
			V248			
			E256			
			M257			
			H259			
			N260			
			H263			
			F264			
			S282			
			V283			
			A323			
	DIP3	−9.3	L63	n.d.	n.d.	N81
			Y65			E189
			K79			V248
			A80			G281
			N81			
			E189			
			H244			
			A246			
			V247			
			V248			
			F264			
			R268			
			L275			
			P277			
			G281			
			S282			
			V283			
			P284			

n.d., not detected.
